# Synthesis and Characterization of Dispersible Geopolymer Nanoaggregates

**DOI:** 10.3389/fchem.2021.751085

**Published:** 2022-05-02

**Authors:** Dinesh Medpelli, Dong-Kyun Seo

**Affiliations:** School of Molecular Sciences, Arizona State University, Tempe, AZ, United States

**Keywords:** geopolymer, nanostructured materials, aggregates, zeolites, zeta potential

## Abstract

We present a simple synthetic route to submicron-sized both potassium- and sodium-based geopolymer nanoaggregates whose nanostructure is suitable for applications in polymer composites. The new synthetic method is based on the chemical mechanism of geopolymer formation in which the extent of cross-liking of geopolymer primary particles is dependent of the alkali concentration and the relative amount of water in the precursor mixture. The products exhibited ∼50–60 nm-sized primary particles along with ∼15–20 nm-sized smaller particles. The external surface areas of the products were high, up to 231 m^2^/g, especially for the sodium-based geopolymer. The primary particles are fused together to form aciniform nanoaggregates with average size of about 400 nm and mesopore volume up to about 0.59 cm^3^/g. The zeta potential of the nanoaggregates was below ‒ 40 mV in the pH range of 5.7–12, demonstrating that the particles are stable in this pH region and do not undergo aggregation and/or agglomeration. All these characteristics make the new material favorable in application of the material in nanofiller application.

## 1 Introduction

Over the last decades, particle-filled or reinforced polymer composites have become very attractive owing to their low cost and widespread industrial applications. Polymer composites are manufactured commercially for a variety of applications such as sporting goods, aerospace components, automobiles, etc. (Hussain et al., 2006) Most usage of elastomers would be impractical without reinforcing them with certain fillers, such as carbon blacks (CBs) and structured silica. Reinforcing silica, either precipitated or “fumed” grades, display intricate assemblies, from primary spherical particles that fuse chemically into aggregates which then form agglomerates linked by weak van der Waals forces. Precipitated silica is most widespread due to the cheaper production costs. It is produced by the controlled neutralization of sodium silicate solution by either concentrated sulfuric, hydrochloric or carbonic acids. Reaction conditions are manipulated according to the particle size requirements (Majumdar, 2005; Schaefer et al., 2010).

Meantime, several grades of precipitated amorphous aluminosilicates have been commercialized by J. M. Huber Corporation under the trademark Zeolex^®^ and Hydrex^®^. They are generally synthesized at room temperature or slightly elevated temperatures by controlled addition of aluminum sulfate (or alum) solution to a solution of sodium silicate while maintaining the pH between 9 and 12. (Bertorelli, 1956; Bertorelli, 1958; Hackbarth and Joseph, 1971) The precipitated aluminosilicate particles are then separated via filtration or centrifugation and purified by repeated washing. These products are currently accepted as being suitable for rubber compounding and paper making. However, one major problem that has been cited is that these products are unstable for long term storage and tend to settle down in strata probably due to gelling of the particles. Strategies to increase their long-term stability have also been proposed. (Shurling, 1966) Furthermore, these products are often contaminated by co-precipitated sulfates (up to 5 wt %) during the synthesis.

Herein, by modifying the chemistry of geopolymerization, we report a unique synthetic approach to produce highly dispersible aluminosilicate particles ‒ dispersible geopolymer particles or simply “DGP” here after. Characterization of the DGP has been carried out with the focus of potential application as nanofillers. Geopolymers are synthetic amorphous aluminosilicates prepared almost all the time in monolithic form but they are innately nanomaterials with a dense xerogel-like microstructure consisting of highly fused three-dimensional network of spherical primary particles of the sizes ranging from 10–50 nm, which are in turn made up of disordered corner-sharing AlO_4_ and SiO_4_ tetrahedra. (Kriven et al., 2003). The synthesis of geopolymers starts with a highly concentrated, viscous aluminosilicate precursor solution (called “geopolymer resin”) with an alkali concentration over 10 *M* and a mole fraction of water less than 0.7. Heating the resin gently at an ambient condition result in hard monolithic geopolymer materials. The amount of alkali (NaOH) used is equal in mole number to that of aluminum (i.e., Na/Al = 1) such that charge neutrality is maintained in the final products. By using excess amount of water (mole fraction of water = 0.73) and alkali (Na/Al =3) we were able to prevent the extensive fusing of primary geopolymer particles and therefore produce DGP which are structurally like CBs and structured silica. In this work, the structure and dispersibility of DGP are investigated and the results are compared with CBs, a reinforcing structured silica and the commercial aluminosilicates, Zeolex^®^ and Hydrex^®^.

## 2 Experimental

### 2.1 Synthesis of Dispersible Geopolymer Particles

Deionized water was used throughout the synthesis and purification. In a typical synthesis, 9.114 g of NaOH pellets (Reagent grade, ≥ 98%, Sigma Aldrich, Product# S5881, Lot# SLBH8376V) and 23.411 g of water glass (∼62.9 wt% H_2_O, 10.6% Na_2_O, 26.5% SiO_2_; Reagent grade, Sigma Aldrich, Product# 338443, Lot# MKBH9050V) were dissolved in 16.382 g of water in a polypropylene beaker. Once cooled down, 11.467 g of metakaolin (MetaMax^®^ from BASF, Lot# 10408G023) (MetaMax PA Technical Bulletin, 2012) with average particle size of 1.3 µm was slowly added into the solution while stirring. The resulting mixture was homogenized with a mechanical mixer (IKA^®^ RW 60 digital mixer) at 800 rpm for about 40 min to obtain a visually homogeneous and free flowing resin (“geopolymer resin”) with the nominal molar composition of 3.0Na_2_O: 1.0Al_2_O_3_: 4.0SiO_2_: 32.4H_2_O.

The geopolymer resins were poured into 50 ml polypropylene centrifuge tubes leaving a headspace of 10–15 ml with a tightly closed lid and the tubes were placed in a laboratory oven at 60°C for appropriate durations. Samples heated at 60°C for 6, 12, 18 and 24 h are denoted as DGP, Z12, Z18 and Z24, respectively. After heating, the loosely aggregated powder products were dispersed in deionized water via homogenization at 6000 rpm (IKA^®^ T25 Digital ULTRA-TURRAX^®^ homogenizer) for about 10 min to give a homogeneous dispersion with a consistency close to milk. It was noticed that the products were ultrafine particles (nanoparticles) which could not be isolated via simple vacuum filtration.

For products purified by repeated washing with water, solids were isolated by repeated centrifugation (4000 rpm or 2156 RCF for 10 min) and redispersion in water until the pH decreased to about 8. For the products purified with an acid wash, ∼2 *M* hydrochloric acid (34–37 wt%, ACS grade, BDH) solution was added dropwise, while stirring with a magnetic stirrer until the pH of the dispersions decreased to about 8. As the pH approached close to 8, solid particles started to precipitate. The solid particles were then isolated via centrifugation (4000 rpm or 2156 RCF for 5 min) and were washed thrice with repeated centrifugation and redispersion in deionized water. Water-washed and acid-washed samples are labelled with the three letter codes as described in previous section followed by “–W” and “–A”, respectively. For example, DGP-W represents a sample prepared by heating at 60°C for 6 h and purified via water washing, and Z12-A denotes a sample prepared by heating at 60°C for 12 h and purified via acid washing. The product precipitates were further treated in three different ways: 1) stored wet at room temperature in tightly sealed polypropylene tubes with added water so that the surface of the products does not dry out upon storing for long periods of time, 2) oven-dried in a laboratory oven at 95°C overnight, and 3) freeze-dried over 2 days and stored in sealed glass vials at room temperature for further analysis.

### 2.2 Materials Characterization

The materials characterizations used conventional procedures for nanostructured metal oxides and the details of the experiments are given so that the characterizations can be performed by following the descriptions. The precipitates could be hand-ground easily. All the characterizations were carried out with a sample finely ground by using a mortar and pestle for 10 min. Powder X-ray diffraction (PXRD) patterns of the finely ground samples were collected using a Siemens D5000 X-ray Diffractometer (Ni-filtered Cu Kα radiation with a wavelength of 1.5406 Å, operated at 40 kV and 30 mA, VANTEC-1 position-sensitive detector) at a scan speed of 2.0°/min and a step size of 0.016° 2*θ*. The resolution of the VANTEC-1 position-sensitive detector was 2*θ* = 0.008°.

Scanning electron microscopy (SEM) imaging of powdered samples was performed with a SEM-XL30 Environmental FEG (FEI) microscope. The analysis was performed with 15 kV acceleration voltage and a spot size of 3 µm. For SEM, finely ground dried sample powders were sprinkled on to the SEM stub affixed with copper conducting tape and the samples were then coated with gold in a sputter coater for 75 s right before imaging. Transmission electron microscopy (TEM) imaging was performed on a JEOL TEM/STEM 2010F (Schottky Field Emission source, accelerating voltage 200 kV). For TEM, the dried powders were quickly sprinkled onto a copper grid that is covered with a holey carbon film and the sample was loaded into the TEM chamber immediately.

Brunauer-Emmett-Teller (BET) surface areas were estimated from gas sorption isotherm analysis by using a Micrometrics ASAP 2020 volumetric adsorption analyzer with nitrogen as the adsorbate at 77 K. Prior to the analysis, samples (about 500 mg) were degassed at 250°C for at least 12 h under vacuum until a residual pressure of ≤10 μmHg was reached. The specific area (SSA_BET_) was calculated according to the BET equation, using nitrogen adsorption isotherms in the relative pressure range from 0.01 to 0.2. (Brunauer et al., 1938). Specific surface area of micropores (SSA_micro_) and the micropore volume (V_micro_) are calculated by applying t-plot method in the thickness range of 0.35–0.50 nm and Harkins and Jura thickness equation. External surface area (SSA_ext_) is estimated as the difference between specific surface areas obtained from BET equation and t-plot method. For the calculation of mesopore size distribution, desorption branch was considered, and the total pore volume (V_total_) was obtained from the amount of nitrogen adsorbed at a relative pressure (P/P_o_) of 0.99, assuming complete pore saturation. Mesopore size distributions were obtained using the Barrett-Joyner-Halenda (BJH) method assuming a cylindrical pore model (Barrett et al., 1951).

Bulk densities of samples were measured for selected products in a pellet form by means of pycnometry using water as a medium, with varied pelletization load and temperature. The pellets were prepared by pressing about 0.2 g of powder in a 10 mm die using a hydraulic press under a pressure of 90,000 psi or 620 MPa. The circular pellets were then heated in air at a designated temperature for 6 h. Elemental compositions and atomic ratios of silicon to aluminum (Si/Al) of the dried products were determined by using Thermo Scientific iCAP 6300 inductively coupled plasma-optical emission spectrometer (ICP-OES). Prior to the analysis, solid samples were acid-digested using a CEM MARS 6 microwave reaction system in repeated heating steps at 180°C for 30 min with sequential addition of required reagents. Specifically, 20–30 mg of catalysts were heated in the reactor first with 3 ml of concentrated HCl solution (34–37 wt%, ACS) and heated again after adding a mixture of 3 ml concentrated HNO_3_ (67–70 wt%, ACS) and 0.5 ml of HF solution (48–51 wt%, ACS). The digests were later quenched with 5 ml of 4.5 wt% H_3_BO_3_ solution and heated in the microwave reactor.

Dynamic Light Scattering (DLS) and zeta potential measurements of the sample dispersions in deionized water at 25°C were performed on Malvern Nano-ZS instrument equipped with a multi-purpose titrator (MPT-2). The wavelength of the laser was 633 nm, and the refractive index of the material was chosen to be 1.47. The concentrations of the aqueous dispersions for DLS measurements were about 50 ppm. The sample dispersions were prepared by hand shaking for 10 s followed by ultrasonicating for 5 min. Titrations were performed on the dispersions between a pH range of ∼12.0 to ∼3.5 below which the aluminosilicate particles can dissolve. Freshly prepared solutions of NaOH (0.01 *M*) and HCl (0.01 *M*) were used to control the pH of the MPT-2 titrator.

## 3 Results and Discussion

**FIGURE 1:**
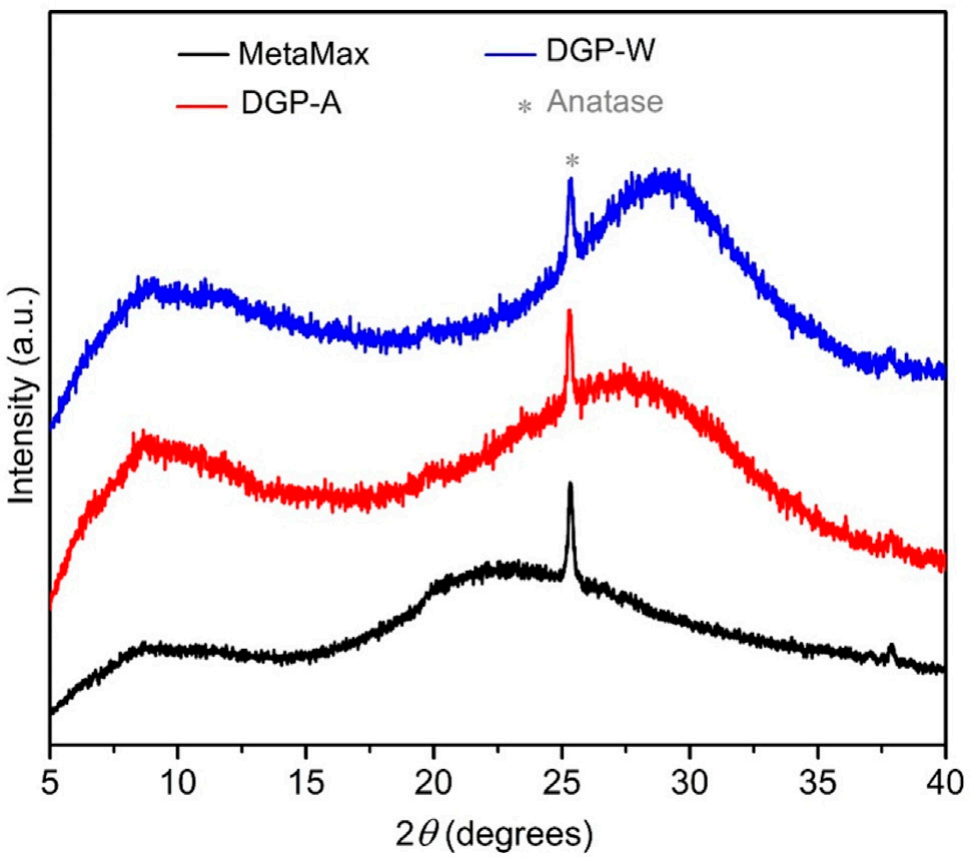
Stacked powder X-ray diffraction patterns of MetaMax^®^ (black) compared with samples DGP-A (red) and DGP-W (blue). Small impurity of anatase (*) (TiO_2_ at ∼25.3° 2*θ*; PDF card # 00-021-1272) is present in all three samples.

**FIGURE 2:**
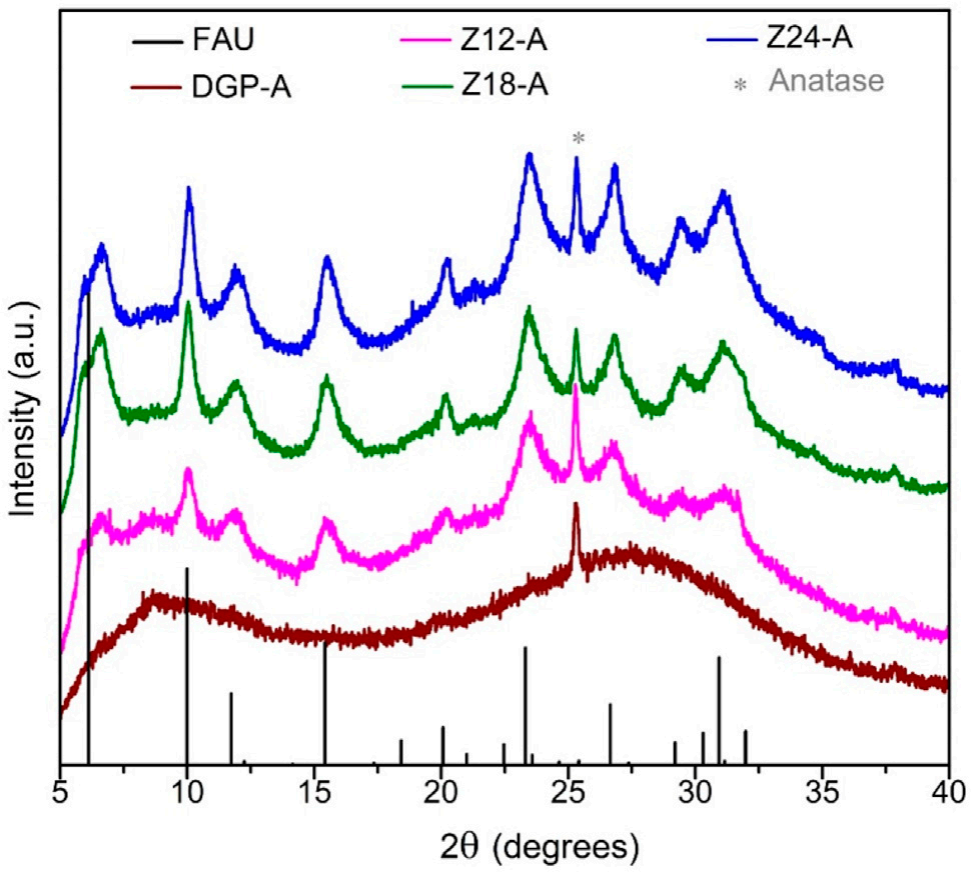
Stacked powder X-ray diffraction patterns of samples DGP-A, Z12-A, Z18-A and Z24-A (bottom to top) compared with the simulated powder pattern of NaX zeolite with faujasite (FAU) structure and (*) anatase (TiO_2_ at ∼25.3° 2*θ*; PDF card # 00-021-1272).

All the products exhibited a light beige color that is identical to the color of the metakaolin precursor (MetaMax^®^). The off-white color is due to amorphous iron oxide impurities that exist in a minute amount in the clay. The Powder X-ray diffraction analysis (Figure 1) confirmed that the metakaolin used in the synthesis is amorphous with a small crystalline peak at ∼25.3° 2*θ* corresponding to TiO_2_ (anatase; PDF card # 00-021-1272) which is present as an impurity. As seen in Figure 1, the broad hump centered at ∼22°2*θ* for the metakaolin is replaced by a new hump centered at 28–30° 2*θ* upon after the chemical reaction (DGP-W and DGP-A), indicating that the products exhibit the amorphous geopolymer structure. It is clear from the powder X-ray analysis of DGP-W and DGP-A samples that different methods of purification (water wash vs. acid wash) had no effect on the crystallinity of the final products. Upon increasing the heating time to 12 h, crystalline peaks corresponding to zeolite with FAU (faujasite) structure started to appear (Figure 2) demonstrating that the onset of FAU crystallization occurred between 6 and 12 h of heating. The percent crystallinity was determined from the micropore surface area with respect to 13X (a commercial FAU obtained from Sigma Aldrich) was 16% after 12 h. Further increase in the heating time to 18 and 24 h caused increment in the crystallinity to 31 and 36%, respectively. The FAU peaks seen in Figure 2 are rather broad signifying the presence of nanocrystals or a poor crystallinity. Beyond 24 h, the crystallinity of the FAU phase did not increase, but instead a competing SOD (sodalite) phase started to appear. Separately, to reduce the synthesis period, heating at 90°C was attempted but crystalline zeolitic phases such as SOD (sodalite), LTA (Linde Type A) and FAU (faujasite) appeared as early as within 1 hour. From these initial studies, it was apparent that heating temperature of 60°C and heating duration of 6 h are appropriate to produce amorphous geopolymer particles i.e., DGP. Since the focus of this work is on amorphous geopolymer particles, the samples DGP-W and DGP-A were further analyzed for their morphologies, dispersibility, and fragility under external pressure.

**FIGURE 3:**
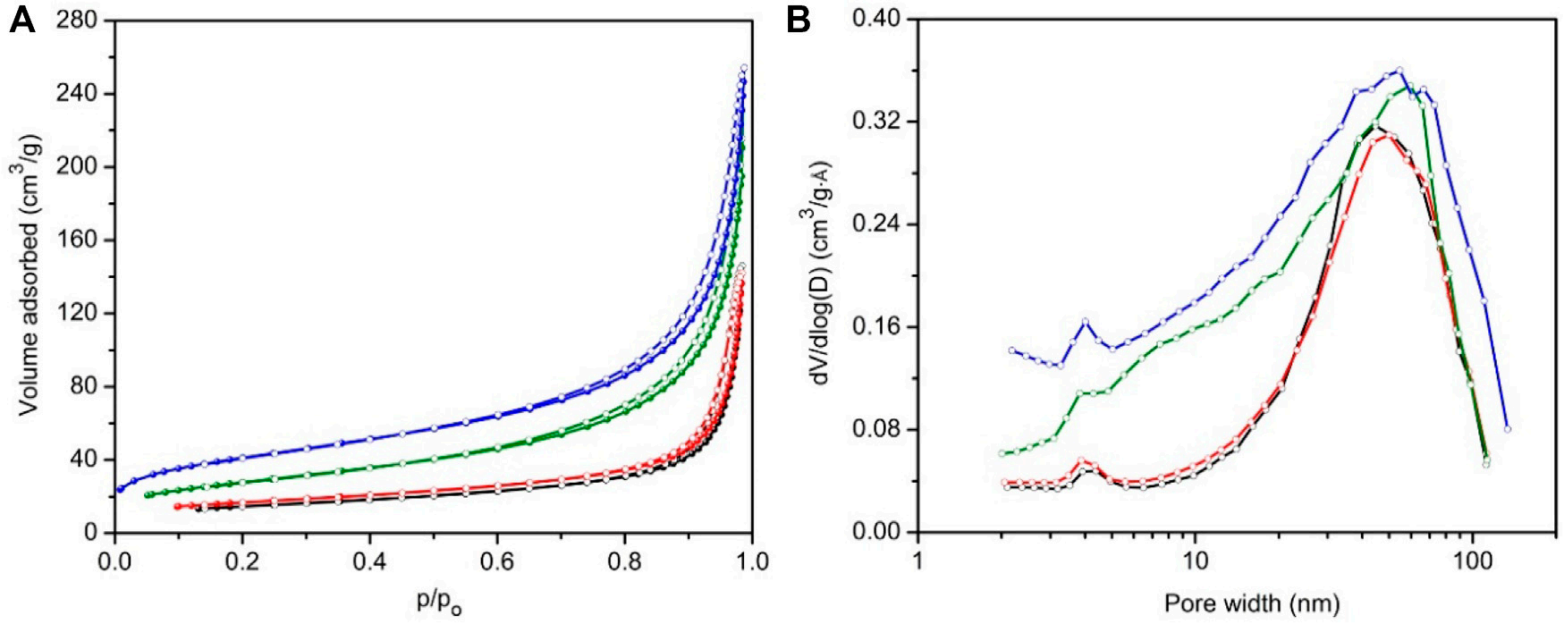
N_2_ sorption isotherms **(A)** and BJH pore size distributions **(B)** of the samples prepared from geopolymer resin heated for 6 h at 60°C, namely, DGP-A-freeze-dried (blue), DGP-A-oven-dried (olive), DGP-W-freeze-dried (red) and DGP-A-oven-dried (black). For all samples, solid spheres and open circles represent adsorption and desorption branches, respectively.

Figure 3A shows the nitrogen sorption isotherms of samples prepared at 60°C, namely, DGP-W and DGP-A, after both oven drying and freeze drying. All samples exhibited type IV isotherms, typical of materials having mesopores. Insufficient N_2_ uptake at low partial pressures ruled out the presence of any micropores or zeolitic phases, which is consistent with the PXRD analysis. Isotherms did not show any signs of saturation at a partial pressure, P/P_o_ ≈ 1.0, indicating co-presence of macropores in addition to mesopores. Furthermore, presence of type H1 hysteresis at high relative pressures (P/P_o_ ≥ 0.6) and absence of saturation at a partial pressure, P/P_o_ ≈ 1, corroborate the presence of mesopores and macropores, respectively. (Rouquerol et al., 1994). Presence of a range of mesopores which extend into the macropore region is clearly seen from their BJH desorption pore size distribution curves shown in Figure 3B. BET surface areas of these samples (listed in Table 1) ranged from 53 to 148 m^2^/g indicating that they are nanoparticulates.

**TABLE 1:** Selected properties of dispersible geopolymer particles obtained from various synthetic conditions.

Sample	SSA_BET_ ^a^ (m^2^/g)	SSA_micro_ ^b^ (m^2^/g)	SSA_ext_ ^c^ (m^2^/g)	V_total_ ^d^ (cm^3^/g)	V_micro_ ^b^ (cm^3^/g)	V_meso_ ^e^ (cm^3^/g)	Average Pore Size^f^ (nm)	PXRD phase	Average Particle size^g^ (nm)
DGP-W									
Oven-dried	53	7	46	0.22	0.003	0.22	17	Amorphous	54
Freeze-dried	60	9	51	0.22	0.004	0.22	14	Amorphous	48
DGP-A									
Oven-dried	101	5	96	0.37	0.001	0.37	16	Amorphous	28
Freeze-dried	148	18	130	0.40	0.007	0.39	11	Amorphous	19
Z12-A (freeze-dried)	340	108	231	0.58	0.05	0.53	7	Amorphous + FAU (16%)^h^	–
Z18-A (freeze-dried)	392	207	185	0.68	0.10	0.58	7	Amorphous + FAU (31%)^h^	–
Z24-A (freeze-dried)	431	236	196	0.70	0.11	0.59	7	Amorphous + FAU (36%)^h^	–

a Pressure range P/Po = 0.05–0.20.

b t-plot method in the thickness range of 0.35–0.50 nm.

c SSA_BET_ – SSA_micro_.

d Single point desorption nearest P/P_o_ = 0.98.

e V_total_ − V_micro_.

f 4(BJH desorption pore volume)/(BET surface area).

g Average size = 6000/(SSA_BET_ × ρ), where ρ = 2.1 g/cm^3^ is the density determined by pycnometry.

h Determined from the micropore surface area with respect to 13X.

**FIGURE 4:**
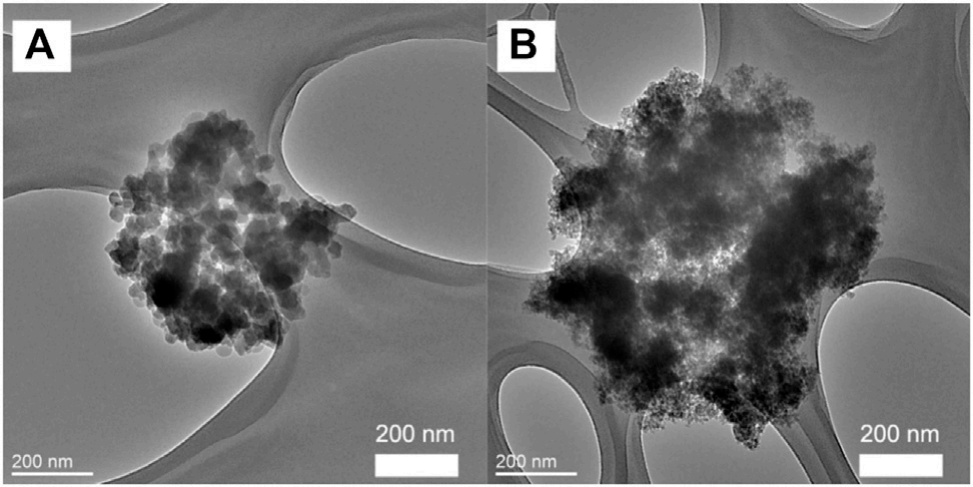
TEM images of amorphous aluminosilicate samples prepared at 60°C for 6 h, 6h-W **(A)** and 6h-A **(B)**.

**FIGURE 5:**
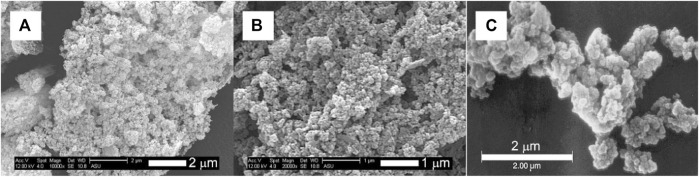
SEM micrographs of DGP-A **(A, B)**, and commercial aluminosilicate, Hydrex^®^ **(C)**, modified from Laine. (Laine)

TEM analysis (Figure 4) revealed that the primary particles smaller than 100 nm are aggregated to form bigger grape-like bundled particles (“primary aggregates”) that are as large as several hundreds of nanometers. Morphologically, these grape-like bundles are similar to carbon blacks with high structure. (Vilgis and Kluppel, 2009) Furthermore, the spaces between the primary particles seen in TEM correspond well with the pore sizes determined by BJH analysis, showing that the meso/macro-pores revealed by N_2_ sorption analysis are inter-particle voids among the primary particles. The SEM analysis (Figures 5A,B) showed that the aggregates are further condensed into bigger micron-sized agglomerates when samples are dried into powders. It is noteworthy that the size of these agglomerates is much smaller in comparison to those observed in the case of Hydrex^®^ (Figure 5C), an aluminosilicate commercialized by J. M. Huber Corporation. (Laine, 2004) Pore volumes ranging from 0.22–0.39 cm^3^/g were estimated from the BJH analysis, while the bulk density of the pellets was determined to be 2.1 g/cm^3^. This means that ∼46–82% of the voids in the pellet are empty space, *i.e.*, the solid fraction of the primary aggregates is quite small (∼0.54–0.18). Relatively big pores (>10 nm) coupled with large void volumes exhibited by the primary aggregates makes these ideally suited as nano-fillers for polymers and paper industry as well as reinforcing rubber.

Table 1 shows the BET surface areas and BJH pore volumes of oven-dried samples of both DGP-W and DGP-A are somewhat smaller than those of freeze-dried samples. This indicates that simple oven-drying of the products during purification leads to strong agglomeration of the primary aggregates when liquid water is driven off via evaporation at high temperatures in a conventional oven, as opposed to removal of frozen water by sublimation and under vacuum in the case of freeze drying. Although two to three times more expensive than traditional drying processes, freeze drying is an industrial process used for drying biomolecules, food, drugs, nanoparticles, etc., where minimal structural distortion upon drying is desired. (Abdelwahed et al., 2006).

**FIGURE 6:**
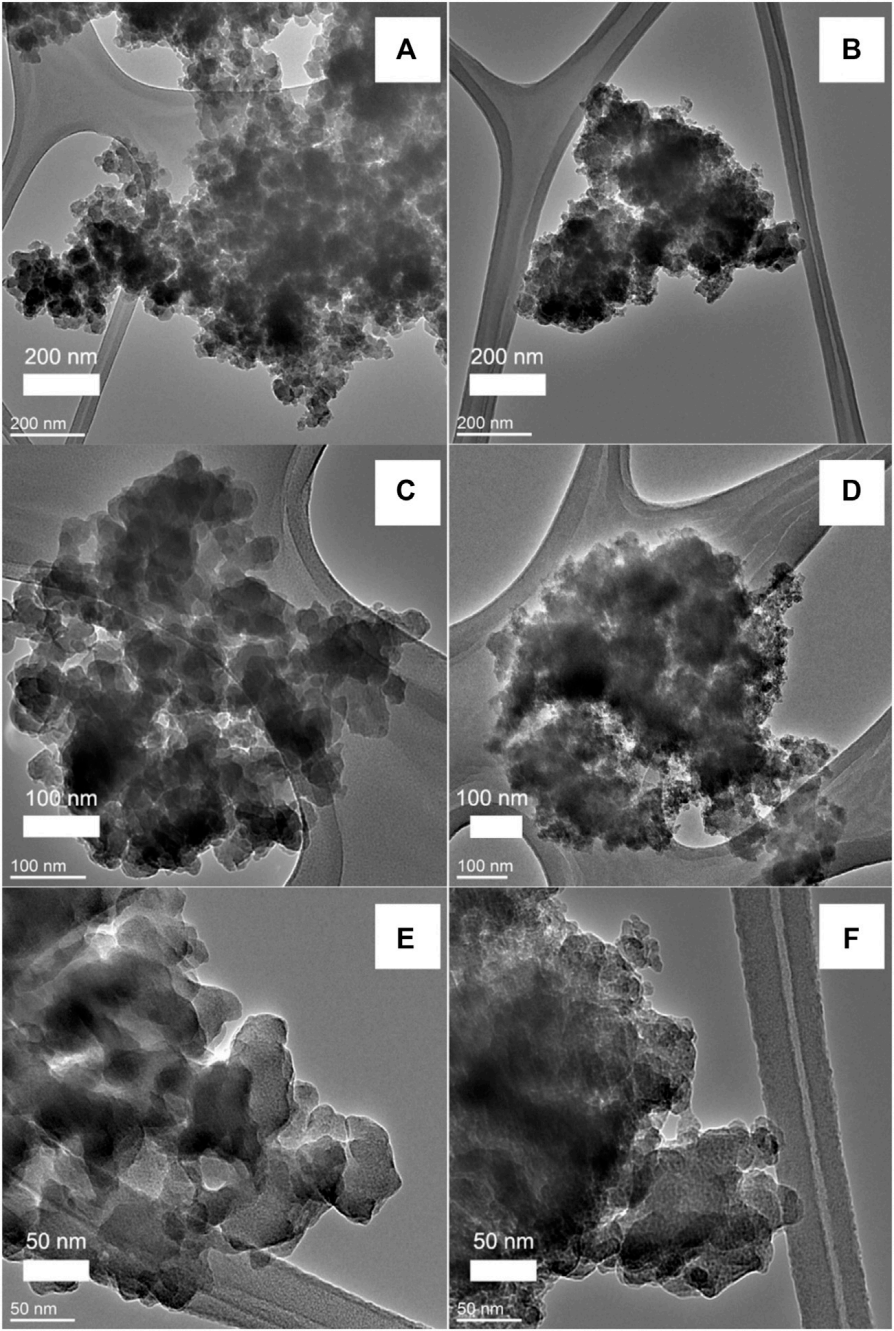
TEM images of freeze-dried samples DGP-W **(A, C, E)** and DGP-A **(B, D, F)** with increasing magnification from top to bottom.

It is also found that acid-washing has an appreciable effect on the morphology of the products when DGP-W and DGP-A are compared. The average particle size of DGP-W estimated from BET surface areas (Table 1) is consistent with those revealed by the TEM studies (Figures 6A,C,E). Meanwhile, there is a disagreement between the particle sizes obtained from BET and TEM analyses for DGP-A. A closer look at the TEM images of DGP-A shown in Figures 6B,D,F reveals that the grape-like aggregates consist of primary particles with two different size ranges, *i.e.*, ∼50–60 nm-sized bigger particles and ∼15–20 nm-sized smaller particles. The discrepancy between BET and TEM could be due to the bimodal particle size distribution, since BET surface area estimates the average size of all the particles put together. Indeed, particle size obtained from BET (19 nm) is in between the bigger and smaller particles.

For the elemental analysis, the ratios between Na and Al were founded to be very close to unity, confirming the successful removal of excess Na^+^ ions upon purification. On the other hand, Si/Al ratio of water-washed DGP-W and acid-washed DGP-A samples was 1.33 and 1.75, respectively, both lower than the nominal ratio of 2.0. It is speculated here that only about two thirds of the nominal silica is incorporated into the geopolymer particles and the rest is present most likely in the form of dissolved silicates. The silicates were washed away gradually during the repeated water washing. Since geopolymers are stable in basic solutions, it is presumed that geopolymer particles resist dissolution during purification and only unincorporated silica is preferentially dissolved and washed away. In the case of DGP-A, however, pH of the dispersion was first dropped to ∼8 upon the acid treatment, and thus unreacted silicates would precipitate out possibly as precipitated silica, (Iler, 1979) before repeated washing with water was performed. Solubility of silica is known to be drastically reduced at pH < 10, which probably led to inefficient removal of unincorporated silica. (Schweitzer and Pesterfield, 2010). It is also reminded that commercial precipitated silica is produced by the controlled neutralization of sodium silicate solution by either concentrated sulfuric, hydrochloric or carbonic acids. Therefore, it is possible that the smaller (∼15–20 nm) particles in DGP-A are mostly precipitated silica. Although not performed, Electron Energy Loss Spectroscopy (EELS) in conjugation with imaging in the Scanning Tunneling Electron Microscopy (STEM) would be one appropriate analytical technique to validate this hypothesis.

**FIGURE 7:**
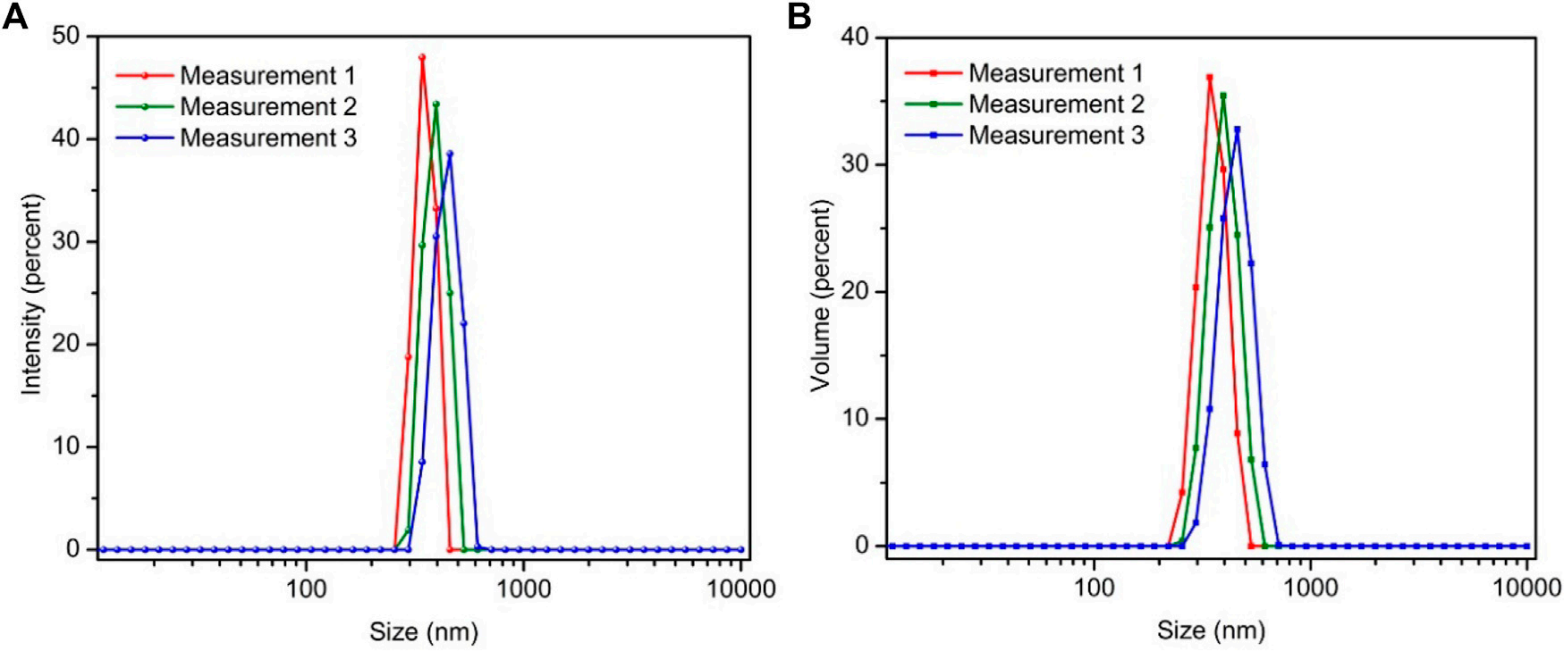
Particle size distribution curves of DGP-W sample by intensity **(A)** and volume **(B)**. Measurements were repeated 3 times in succession.

It is noted that the wet pastes of DGP-W and DGP-A after the centrifugal purification contained about 78 wt% water. Even after long term storage, samples could be readily re-dispersed into fluidic dispersions upon dilution with deionized water (say 10 wt%) and stirring by hand with a metal spatula for approximately 20–35 sec, followed by manual agitation for further 15 sec. This observation demonstrates that DGP particles are stable with long shelf-life and do not gel upon long term storage, unlike Zeolex® and Hydrex® aluminosilicate particles. (Shurling, 1966). Figure 7 shows the particle size distribution of ∼50 ppm dispersion of DGP-W after 10 min of ultrasonication to break up the loose agglomerates. Single peaks with rather narrow distributions were observed. In addition, resemblance of size distribution curves by intensity (Figure 7A) and by volume (Figure 7B) strongly indicate that the bigger particles (seen under SEM) exist only in a minute quantity. This reasoning is from the fact that size distribution by volume is much more sensitive to the presence of even a few bigger particles than does the size distribution by number. The average particle size from the DLS peaks was calculated to be 399 ± 59 nm with a FWHM (full width at half maxima) of 142 ± 33 nm. Those average particle size values correspond well with the sizes of grape-like bundles observed under TEM (Figure 6B). SEM, TEM and DLS results strongly suggest that micron-sized particles that exist in the powder form are loose agglomerates that readily break-up into finer aggregates (hundreds of nm) upon dispersing the powders in water. The aggregates are in turn made-up of strongly glued primary particles (50–60 nm) that could not be broken even after ultrasonication.

**FIGURE 8:**
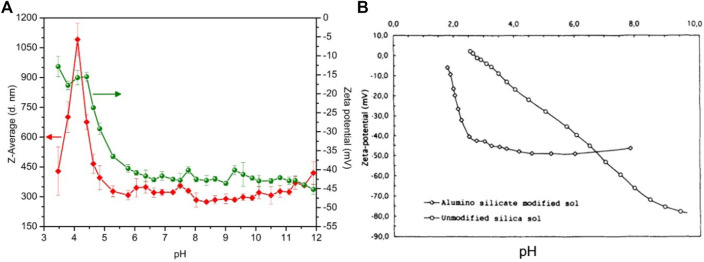
Particle size (red solid diamonds) and zeta potential (green solid spheres) of DGP-W as a function of pH, from this study **(A)**, and zeta potential of unmodified (black open circles) and aluminosilicate modified silica sol (black open diamonds) as a function of pH **(B)**, modified from Otterstedt (1998).

Stability of the particles was studied under acidic and basic conditions. Both zeta potential and particle sizes of DGP-W sample was monitored as a function of pH, as shown in Figure 8A. Zeta potential of DGP-W remained highly negative (absolute value > 40 mV), and the particle sizes remained unaffected (320 ± 31 nm) in the pH range of 12.0–5.7, indicating that the particles are stable in this pH region and do not undergo agglomeration. Below pH of 5.7, The gradual decrease in the zeta potential was found down to the pH value of 4.0 and it was accompanied by a steady increase in the particle size to a maximum of 1091 ± 81 nm at pH = 4.0. This suggests aggregation/agglomeration in this region. An unusual trend observed in both zeta potential and particle sizes below pH 4.0 could be because aluminosilicates undergo dissolution under exceedingly acidic conditions. A similar observation has been reported for aluminosilicate modified silica sols. (Otterstedt, 1998). On the other hand, the zeta potential of silica progressively decreases in the entire pH region of 10.0 to 3.0, signifying their instability compared to aluminosilicates (Figure 8B).

Since the compounding of fillers with a polymer melt (rubber for tire, for example) is done under a high pressure, it is essential for the nanoparticulate fillers to withstand high pressures. To test the tolerance of the DGP aggregates to high pressures, both oven and freeze-dried DGP-A samples were pressed into circular pellets at 90,000 psi or 620 MPa and their pore properties were measured employing N_2_ sorption analysis. Specific surface areas, pore volumes and average pore widths before and after pressing pellets are compared in Table 2. Although the surface area and pore volume, as well as average pore size to a lesser extent, decreased for both the samples upon pelletizing as one would expect, more than 60% of the porosity in these samples is retained, indicating that the samples do not crumble completely under the extreme pressure of 620 MPa. It is worth mentioning that the typical industrial standard procedure (ASTM D3493) of measuring a materials resistance to pore collapsing is performed under a pressure of only 165 MPa, (George et al., 2011) which is 3.75 times smaller than the pressure used in this study.

**TABLE 2:** Pore characteristics of selected sample pellets subjected to various heat treatments.

Sample	Sample Form	Temperature (°C)	BET Surface Area^a^ (m^2^/g)	Pore Volume^b^ (cm^3^/g)	Average Pore Size^c^ (nm)	Average Particle size^d^ (nm)
DGP-A (freeze-dried)	Powder	25	148	0.39	11	22
	Pellet	25	82	0.21	10	35
	Pellet	400	72	0.25	14	40
	Pellet	500	63	0.23	15	48
	Pellet	600	32	0.22	28	102
	Pellet	700	4.9	0.05	41	752
DGP-A (oven-dried)	Powder	25	101	0.37	16	30
	Pellet	25	54	0.30	15	57
	Pellet	400	52	0.29	22	58
	Pellet	500	47	0.25	23	65
	Pellet	600	39	0.23	25	75
	Pellet	700	15	0.11	34	204

a Pressure range P/Po = 0.05–0.20.

b Single point desorption nearest P/P_o_ = 0.99.

c 4(BJH desorption pore volume)/(BET surface area).

d Average size = 6000/(SSA_BET_ × ρ), where ρ = 2.1 g/cm^3^ is the density determined by pycnometry.

Sintering properties of the materials were also evaluated by subjecting the pressed pellets to heat treatment at several different temperatures and measuring their pore properties after each subsequent heating step (Table 2). After heating, all the pellets showed a shiny, uniform surface to a naked eye, but surface cracks were noticeable under an optical microscope. The surface area of both freeze-dried and oven-dried samples decreased from 82 m^2^/g to 4.9 m^2^/g and from 54 m^2^/g to 15 m^2^/g, respectively, upon heating at 700°C, as expected due to the sintering of nanoparticles and therefore pore collapsing at elevated temperatures. For most of the samples, the surface area decrease was accompanied by a gradual decrease in the pore volume upon increasing the heating temperature. However, the freeze-dried DGP-A showed a slight increase from 0.21 cm^3^/g to 0.25 cm^3^/g after heating at 400 °C for 6 h. This unusual increase can be understood on the basis that as the particles get sintered, pores get wider and the slight increase in pore volume might be because of the presence of the wider pores. In any event, a clear trend of increasing average pore widths is seen with increasing temperature for both freeze-dried and oven-dried DGP-A samples, confirming the pore collapse. Although not shown here, even after heating at 700°C for 6 h, samples were still amorphous with no hint of crystallization or structural change observed by powder X-ray diffraction (PXRD) measurements. This observation is in line with literature i.e., geopolymers do not crystallize below 1000°C. (Davidovits, 2011). This heat treatment study demonstrated that DGP is suitable for high temperature applications.

## 4 Conclusion

We have demonstrated that it is possible to synthesize highly dispersible geopolymer/ amorphous-aluminosilicate particles by a simple modification of the geopolymerization process. The simplicity of the modified geopolymerization process bodes well for the large volume production of DGP. By means of nitrogen gas sorption, SEM and TEM, we characterized the morphology of DGP to be grape-like bundles similar to high structure CBs and structured silica. Like the latter, grape-like bundles of DGP are fused into loosely held agglomerates, as revealed by SEM and DLS studies. The large surface area and high structure, DGP has a potential to be used as a reinforcing filler. Moreover, highly negatively charged surface of the DGP in a wide pH region is unique, making it a good candidate for surface modification in order to increase filler-matrix compatibility. The off-white color of DGP can only expand its potential as a reinforcing agent in polymeric applications, as well as a pigment in paper industry, for example. Importantly, unlike commercial aluminosilicates such as Zeolex^®^ and Hydrex^®^, pastes and dispersions of DGP do not undergo gelation upon long term storage. We also demonstrated that DGP are stable in a rather wide pH range of 12.0–5.7 below which gradual agglomeration was noted. In addition, DGP powders are shown to withstand large external pressures indicating that the pressures used during compounding to make composites would not be problematic. Further studies are desired to design a better purification process than time and energy intensive centrifugation reported here.

## Data Availability

The original contributions presented in the study are included in the article/Supplementary Material, further inquiries can be directed to the corresponding author.
